# Developed-developing country partnerships: Benefits to developed countries?

**DOI:** 10.1186/1744-8603-8-17

**Published:** 2012-06-18

**Authors:** Shamsuzzoha B Syed, Viva Dadwal, Paul Rutter, Julie Storr, Joyce D Hightower, Rachel Gooden, Jean Carlet, Sepideh Bagheri Nejad, Edward T Kelley, Liam Donaldson, Didier Pittet

**Affiliations:** 1 African Partnerships for Patient Safety, WHO Patient Safety, WHO Headquarters, Avenue Appia, 1211, Geneva 27, Switzerland; 2 Infection Control Programme and WHO Collaborating Centre on Patient Safety, University of Geneva Hospitals and Faculty of Medicine, 4 Rue Gabrielle Perret-Gentil, 1211, Geneva 14, Switzerland; 3 National Patient Safety Agency, 4-8 Maple Street, London, W1T 5HD, United Kingdom

**Keywords:** Developed countries, Developing countries, Partnerships, Learning, International cooperation, Health care quality, Global health

## Abstract

Developing countries can generate effective solutions for today’s global health challenges. This paper reviews relevant literature to construct the case for international cooperation, and in particular, developed-developing country partnerships. Standard database and web-based searches were conducted for publications in English between 1990 and 2010. Studies containing full or partial data relating to international cooperation between developed and developing countries were retained for further analysis. Of 227 articles retained through initial screening, 65 were included in the final analysis. The results were two-fold: some articles pointed to intangible benefits accrued by developed country partners, but the majority of information pointed to developing country innovations that can potentially inform health systems in developed countries. This information spanned all six WHO health system components. Ten key health areas where developed countries have the most to learn from the developing world were identified and include, rural health service delivery; skills substitution; decentralisation of management; creative problem-solving; education in communicable disease control; innovation in mobile phone use; low technology simulation training; local product manufacture; health financing; and social entrepreneurship. While there are no guarantees that innovations from developing country experiences can effectively transfer to developed countries, combined developed-developing country learning processes can potentially generate effective solutions for global health systems. However, the global pool of knowledge in this area is virgin and further work needs to be undertaken to advance understanding of health innovation diffusion. Even more urgently, a standardized method for reporting partnership benefits is needed—this is perhaps the single most immediate need in planning for, and realizing, the full potential of international cooperation between developed and developing countries.

## Background

International cooperation is crucial for improving global health outcomes. One such form of cooperation occurs through international partnerships, which lead, stimulate, and facilitate action on health challenges through programming, advocacy, and technical support. Just as the preference for the term ‘global health’ has increased [1], so has the shift in philosophies and attitudes to partnership-building. Partners today increasingly seek mutuality of benefits, including two-way flow of energies, expertise, and knowledge to justify investment.

At the same time, more and more health leaders are turning their attention to developing countries to generate effective solutions for health [2-6]. One such leader is Lord Nigel Crisp, the former Chief Executive Officer of the U.K. National Health Service, who states, “rich countries can learn a great deal about health and health services from poorer ones…combining the learning from rich and poor countries can give us new insight on how to improve health” [2]. The private sector has already embraced the sensation—termed ‘reverse innovation’—and corporations are rapidly promoting the spread of developing country innovations worldwide [3].

African Partnerships for Patient Safety (APPS) is a WHO programme that has built patient safety partnerships between hospitals in Africa and Europe. Partnership strengthening is a core APPS programme objective [7] and building a business case for international cooperation (in particular, developed-developing country partnerships) is a critical component of this objective. This interest informed the main purpose of our research. In this paper, we relay existing information on health system benefits accrued by developed countries from partnering with developing countries, and then gauge whether developing country health system experiences could form the basis of future international cooperation.

## Methods

### Search strategy and selection criteria

Five MeSH search headings, “health care quality”, “access and evaluation”, “international cooperation”, “hospitals”, and “learning”, were combined and exploded with geographical qualifiers such as “Africa” OR “Asia” AND “Europe” OR “North America” to identify studies with full or partial information related to developed-developing country cooperation. Developed and developing countries were defined according to the 2008 World Bank classification [8]. English language articles between 1990 and 2010 were included and searches were carried out on PubMed, Google, and grey literature databases. Relevant articles were retrieved and their reference lists searched for additional articles. Abstracts were evaluated for their suitability to the research question. For each article included, the reviewer completed a data extraction form to summarize key details (i.e., author, year of publication, key points, and health/clinical benefits). Relevant articles were appraised for inclusion by one investigator (VD) and confirmed by a second investigator (SBS). Disagreement was resolved by consensus. The information searching and extraction process was iterative. The comprehensive search strategy and the appraisal questions are provided separately in Appendix 1/Additional File 1.

**Figure 1:**
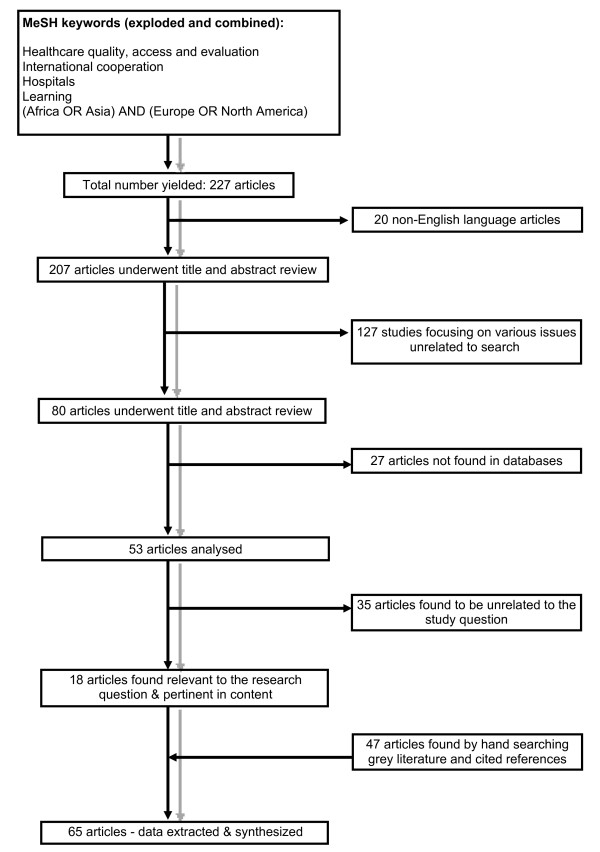
Flow chart for selection of articles.

Our search yielded 227 articles, of which 18 were eligible for inclusion. Relevant articles and their bibliographies generated new leads, which were also evaluated for inclusion. A total of 65 articles were finally included in the review (see Figure 1 for more detail).

## Results

**Figure 2:**
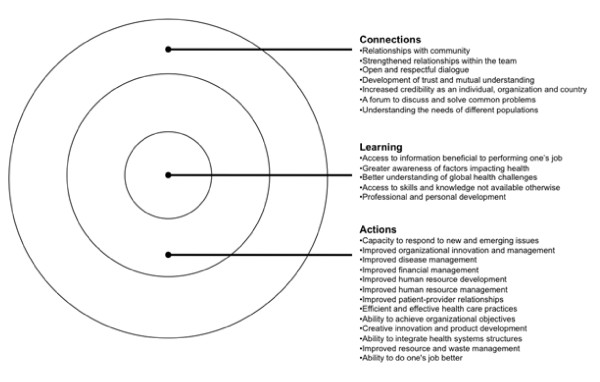
Benefits to individuals and organizations in developed countries.

Benefits accrued by developed countries from partnering with developing countries were found to principally span the first three intangible, or ‘soft’, elements of the Partnership Evaluation Tool (PET), a model that identifies four categories of partnership benefits, namely ‘connections’, ‘learning’, ‘action’, and ‘impact’ [9]. These benefits predominantly influence health workforce education and training and include examples such as improved employee morale, heightened learning, better information sharing, personal and professional development, improved patient-provider relationships, and a greater awareness of the factors impacting health (see Figure 2 for more detail). We did not find clear evidence for the broader ‘impact’ of such benefits on developed country health systems, perhaps because soft benefits are difficult to measure and their effects hard to trace [10].

Rather, our research overwhelmingly detected unique ways of responding to health challenges that support the concept of reverse innovation. Narrative summaries describing our main findings are provided below, arranged according to the six key components of a health system [11]. These accounts provide insights on how innovations (e.g., a new method, idea, product, policy, etc.) in developing countries can inform responses to contemporary health system challenges in developed countries (see Table 1).

**Table 1:** Summary of key system-wide benefits arranged by the six WHO building blocks of health systems

Service delivery	· Developing countries provide examples of effective, safe, good quality personal and non-personal care
	· These examples include service priorities like integrated delivery packages; delivery models; infrastructure and logistics of the provider network; leadership and management; safety and quality; and demand for care
	· The organization and management of inputs and services to deliver such packages of care can offer insights to the developed world
Health workforce	· Developing countries have found unique and innovative ways of dealing with the human resource crisis in health and can provide developed countries with ideas on how to best mobilize workers and fill gaps in skills and shortages
	· These insights include lessons on planning and scaling-up work force; designing training programmes for integrated service delivery; organization of healthcare workers; and recruitment and retention of workers
Health information	· Developing countries are excelling in the production, analysis and dissemination of information using mobile technology - this platform is usable on a regular basis and in emergencies
	· Developing countries are supporting the development of national information systems; robust reporting and response mechanisms; and the ability to track health system performance through e-health information systems
	· Developing country experiences and successes in mobile health technology utilization can inform and stimulate patient-centred care in developed countries
Medical Products, Vaccines, and Technologies	· A number of developing country innovations aim to ensure equitable access to essential medicinal products
	· These innovations result from frugality rising in resource-poor environments
	· Professionals who are able to create, appreciate, and utilise such innovative products are an asset to their host institutions
Health Financing	· Some developing countries offer health financing systems that achieve universal coverage through social protection
	· These systems provide health for all through national health financing policies; tools and data on health expenditures; and policies on costing/expenditure
	· Despite their differences, developed countries can learn about social protection in health from developing countries
Leadership and governance	· No global blueprint for health leadership and governance exists
	· Developing country leadership and governance examples from successful initiatives (involving policy guidance; collaboration and coalition building; and harmonization & alignment) offer learning opportunities for developed countries

### Service delivery

Many developing countries have developed mechanisms to reduce cultural, social, financial, or gender-related barriers to service delivery [12,13]. For example, family and community-based interventions in developing countries have been indispensable to the management and treatment of diseases like schizophrenia, through de-stigmatizing practices like therapeutic optimism, extended support networks, and more holistic appraisals of the disorder [14,15]. Where direct interaction between men and women is discouraged (e.g., rural communities), gender mainstreaming has allowed for the delivery of gender-specific care through the use of female workers [16]. An Iranian *thalassaemia* prevention programme demonstrates culture-sensitive ways of prevention in high-risk individuals by screening school children as part of their regular health check-ups [17]. Such strategies can be helpful in managing developed-country healthcare challenges in marginalized developed country populations. Indeed, this was the experience for Project Connect, a U.S.-based AIDS treatment program that found inspiration from a similar program in Zambia [18].

Multiple innovative and efficient models for depression care exist in China, Iran, and Tanzania, which integrate mental care into general medical settings through the use of ‘village health workers’ and ‘health houses’ where suitably trained health personnel serve general medical and psychiatric needs of the communities they represent [19]. Developing countries have also long addressed the use of alternative medicine through policy models where modern and traditional medicine are either integrated through medical education and practice, or practiced through parallel mechanisms within the national health system [20]. As Western governments grapple with medical pluralism, developing country models of integrated health can offer guidance on how to provide a care continuum that enhances social integration.

Developing countries can also offer learning opportunities to those seeking to maximise health service coverage, quality, and safety. For example, organisational innovation and management using discriminatory service provision, fixed price models, and efficient supply and delivery chains, has helped improve production efficiency in India [21]. In Ethiopia, quality of hospital care was improved through partnership-mentoring models, which provide new approaches for increasing management capacity and improving hospital management systems [22]. Similarly, task shifting, group interventions, and pricing/procurement strategies in multimodal stepped-care programmes in Chile have produced quality clinical outcomes at low cost [23].

### Focused example: Chronic care models

When doctors running an AIDS clinic in the U.S. sought to increase patient follow-ups, they turned to an unusual place for help: Zambia. Project Connect, a program based in the University of Alabama is reducing patient no-show rates by taking a community and patient centred approach adapted from lessons in Zambia. The Wall Street Journal reports, “Patients were given appointments with doctors within five days of calling the clinic. Blood tests were taken during the first visit. A social worker did an interview, trying to identify and address any issues that might prevent patients from coming back. The no-show rate dropped from 31% in 2007 to 18% through June 2009.” Effective chronic care delivery is possible in resource scarce settings, and developing country models can offer opportunities for combined learning.

Medical tourism has resulted in the rapid development of major teaching hospitals abroad. The work of subsidiary organizations like Harvard Medical International and Johns Hopkins International demonstrates strategic, financial, research and marketing-related opportunities in emerging economies like India, China, Pakistan, Malaysia, Chile, Peru, and Mexico [24]. Several U.S. hospitals are also engaged in hospital and institutional partnerships in countries like Saudi Arabia, India, and Turkey to boost revenues at home [25]. Commercial partnerships can provide a channel to inform key service delivery challenges, including the training and retention of workers, maintenance of quality outcomes, and access to health care.

### Health workforce

Developing countries are promoting novel approaches to dealing with the global shortage of professionally trained healthcare personnel by scaling-up modified service delivery models and introducing specialized worker education and training. For example, several developing countries are effectively and efficiently training mid-level workers to perform emergency interventions [26-29]. In Mozambique and Bangladesh, this approach has generated novel applications of task shifting that has improved health access [30,31]. In Ghana, pre-hospital trauma training of lay workers, such as commercial drivers, has led to a significant reduction in road traffic deaths [32]. In Nepal, the facilitation of professional relationships between traditional healers and government health workers is improving health service delivery [33]. Without doubt, human resource planning and development vary between developed and developing countries. However, as the developed country health workforce diversifies in remote and rural areas, workforce planners may benefit from utilizing developing country models for worker substitution, mobilization, recruitment, and retention.

Community health workers (CHWs) are often underutilized in developed countries and a key challenge remains in institutionalizing and mainstreaming community participation. Training programs involving CHWs and volunteer networks in countries like India [34], Peru [35], Haiti [35], and Brazil [36] have shown tremendous success in improving health outcomes for the chronically ill and dying. Pakistan’s Lady Health Workers is an accredited community-based programme that has improved both the quality of care and gender-based workforce participation [37]. These health-care workers are trusted members of society and play an important role in linking formal health systems to rural communities. Lessons from developing country experiences can help expand the knowledge base on community workforce policy, training, and education, as well as help raise the profile of community-based interventions.

### Focused example: Community health workers

Pakistan’s Lady Health Worker Programme was established in 1994 to provide essential primary health services in rural and urban slums. Over 100 000 lady health workers have been deployed to date, reaching out to over 90 Million Pakistanis in all 135 districts of the country. The Programme is widely regarded as one of the best community-based programmes in the world. Melinda Gates writes about community-based interventions: “By empowering the community with knowledge about lifesaving methods such as skin-to-skin care and immediate breastfeeding, the project cut the mortality rate for newborns in half in only 16 months without introducing any new technology.” Community health workers can play a vital and effective role in a country’s regular health system by improving community self-sufficiency, fostering meaningful use of social systems, and improving health and well-being in neighbourhoods with complex needs. Developing countries have a rich history of both small and large CHW programmes that can offer a source of learning for developed countries wishing to employ such programmes in both rural communities as well as inner-city neighbourhoods.

Finally, clinical and public health training in developing countries can serve as a critical component of skills development and maintenance for developed-country practitioners who are at risk of losing knowledge due to differing patterns of disease burden [38]. Such training allows exposure to a range of diseases, organizations, and management styles, which are necessary to solve today’s global health challenges [39-41]. Partnership reports also describe developing country settings as optimal grounds to build training competencies in the areas of public health policy and administration [42,43]. Some developing country models of patient-centred care give a human face to pathology, priming healthcare workers for expressive relationship-centred care that improves doctor-patient relationships and satisfaction [44]. Finally, connected health models and hospital-to-hospital links such as teleradiology partnerships illustrate how the use of global staffing models can allow for shift flexibility, sub-speciality consultations, and reduced overhead costs [45]. Such links also allow opportunities for practitioner learning through continued professional development [46].

### Health information

Health information technology (IT) and connected health programs are increasingly being leveraged to manage chronic illnesses, maintain health and wellness, improve adherence, engagement, and clinical outcomes in developed and developing countries alike. The rapid expansion of mobile health (or mhealth) in developing countries has created innovation hubs in countries like Kenya [47], Uganda [47,48], South Africa [47,48], Rwanda, [48], and India [48] where mhealth campaigns show high levels of popularity among physicians, and are transforming rural healthcare through improved data collection, disease surveillance, post-discharge surveillance, health promotion, diagnostic support, disaster response, and remote patient monitoring [47-49]. A Ghana-based network called mPedigree is an excellent example of how local IT innovation can protect the lives of people across continents [49]. While developed countries are more likely than developing countries to have a national mhealth policy or strategy, developed country organizational culture remains either unaccustomed to, or hesitant of, the advantages of mhealth [50]. Developing country experiences can promote concerted advocacy efforts on the benefits of mhealth in developed countries, especially for remote patient monitoring, emergency health management, medical adherence, and health education for disadvantaged communities [50].

### Focused example: Health technology and medication safety

Counterfeit pharmaceuticals are being combated through health technology in Africa. mPedigree, an African social enterprise network, provides a mobile phone service which delivers services targeting counterfeit pharmaceuticals in Ghana and Nigeria (an example of very low-tech solution to very major problem). Users simply send a free text message with verification code to one of mPedigree’s partners in Europe for an instant response regarding their medications. mPedigree’s business interfaces also allow pharmaceutical companies to monitor presence of genuine and counterfeit drugs. The program is built on cloud-based technology, scalable infrastructure expandable to other regions. While counterfeit medicines are not nearly as serious in industrialized countries due to safety and quality mechanisms, innovative platforms such as mPedigree provide quick and effective user-based solutions to protect one’s health. Such technologies and business models can be scaled up through international cooperation to more effectively battle the global trade in counterfeit medicines, as well as tackle other medication safety issues such as remote support for aging populations.

### Medical products, vaccines, and technologies

Despite constraints, developing countries produce efficient and effective substitute health products and treatments [3,51-54]. Resource frugality not only compels creativity but also provides the right settings to train employees to adapt, create, appreciate, and utilise health products [54-57]. Indeed, there are numerous cases of famous innovative health products being generated this way. For example, in Ecuador, a simple polymerase chain reaction-based assay was used to diagnose *leishmaniasis*[58]. In Bangladesh, homemade spacers became a cost-effective and quality-assured means to help manage asthma in children [59]. Oral rehydration therapy, a simple treatment developed in Bangladesh to treat diarrhoea using sugar and salt solutions, has now saved millions of lives across the world [60]. Perhaps equally renowned, Kangaroo Mother Care is another example of how simple interventions can be scientifically sound low-cost alternatives [61]. One U.K. doctor, faced with several cases of clubfoot in resource-restricted Malawi, made use of a method devised in the USA in the 1960s called the Ponseti treatment. This treatment, which involves manipulation and splinting, was found to be more simple and effective than surgery and is now popularly used in the USA and Europe [62]. Clearly not all developing country innovations can be adaptable to developed country scenarios. However, a shift of thinking through the reverse flow of knowledge can help bridge the large gap between developed and developing country health products to pave the way for future collaboration.

### Focused example: Kangaroo Mother Care

*In the 1970s, hospitals in Bogota, Colombia, did not have enough incubators to treat premature and low-birth weight infants. Rey and Martinez developed a conceptually simple and elegant intervention out of this scarcity, which relied on continuous skin-to-skin contact between mother and infant. Known as Kangaroo Mother Care (KMC or skin-to-skin care), this intervention quickly became an ideal model for homecare for low-birth-weight infants. Detailed recommendations for the application of KMC have been issued in both developing and developed countries. Enhanced practice of KMC (including continuous skin-to-skin contact) is necessary to reap the benefits of the intervention (*e.g.*, emotional regulation, increased nursing rates and lactation, improved sense of parenting, breastfeeding support, and early discharge) in developed countries. Indeed, this innovation provides high quality care in high-income settings based on an intervention generated for low-income health systems.*

### Financing

Countries have a number of financing strategies at their disposal to advance their health systems. Comparing health-financing reforms between countries is therefore particularly challenging and depends on the ‘starting point’ of each country [63]. That being said, some developing countries have employed innovative financing strategies with a careful choice of well-aligned policy instruments. For example, to avoid direct payments and extend coverage to hard-to-reach groups, Mexico’s public insurance scheme relies on the contributions of the federal government, states, and individuals alike [64]. Similarly, in Colombia, tax-based insurance schemes target both the rich and poor, working hand-in-hand to provide the basic level of care by increasing coverage and service [11]. District health planning matched by targeted incremental budgetary increases have led to a substantial decline in infant mortality and improvement in adult health in Tanzania [65]. Thailand’s tax financed universal coverage reforms in 2001 also reduced supply-side subsidies in favour of a national pool of funds that would ensure inexpensive and easy physical access to services for all [66]. Finally, although debates around microfinancing continue, the Grameen Bank experience in Bangladesh has inspired many countries to pursue economic and social grassroots’ development [67]. It is expected that many developed countries will need to raise additional funds to meet future health demands, particularly due to population aging and the rising costs of new medicines and technologies [68]. Assessing experiences from low- and middle-income countries is not meant to yield strong conclusions about any one particular financing scheme but can help draw lessons for policy-makers who seek ideas on resource diversification.

### Focused example: Microcredit

Born as a social experiment in Bangladesh, the Grameen Bank today serves more than 7 million poor families with loans, savings, insurance and other services. The bank is owned and operated by its clients and has been a model for microfinance institutions around the world. Health-related services have been packaged with these micro-finance initiatives across the world and can have direct positive impacts. Indeed, poverty alleviation is clearly linked with improving a key wider determinant of health. While micro-finance is not a panacea for health financing, the principals used and the experiences (both positive and negative) can inform local approaches to health solidarity.

### Leadership and governance

A growing number of developing country success stories illustrate the progress of global health: polio eradication is closer than ever [69]; low-cost cataract treatments are restoring sight in India [69,70]; simple salt fluoridation has led to significant prevention of dental caries in Jamaica [69]; regional initiatives are succeeding in curbing Chagas disease in South America [69]; oral rehydration therapy has helped reduce infant diarrhoeal deaths worldwide [69]; tuberculosis prevalence is dramatically decreasing in China [69]; and prevention of HIV and sexually transmitted infections in Thailand has led to significantly fewer new cases of HIV [69,71]. Interventions like these would not have been possible without political and community vision and leadership [71-73], resourcefulness, and optimism [74]. Qualitative health-related action research on the Philippines health reform confirms the importance of local solutions to lead the way [75]. Indeed, for many of these successes, domestic health stewardship allowed effective oversight, performance monitoring, coalition building, system design, and accountability.

### Focused example: Catalysing local system performance through leadership

Some leadership programs in developing countries are demonstrating links between transparency, governance, and health outcomes by improving health system capabilities. One such example is provided by the State of Ceará in Brazil, which mandates that public servants receive leadership training to apply for management positions. By weaving leadership development into all underlying talent management systems and processes, the State has been able to strengthen leadership and management of public sector employees. This has contributed to improved health system performances over time. For example, 25 municipalities in Ceará (out of 37) reduced infant mortality between 2000 and 2004—some by as much as 50%. Given that effective leaders and managers lie at the foundation of good governance, identifying key ingredients of successful leadership programmes remains in the interest of developed and developing countries alike. Taking stock of collaborative initiatives to reflect on strengths and weaknesses of such programmes is necessary to seize future opportunities for cross-fertilization of ideas focused on change.

Leadership development continues to be a top strategic priority for senior health leaders around the world, yet most health systems have underdeveloped leadership and management skills and a high rate of turnover in central positions [76]. Furthermore, there is little consensus on how to adequately monitor and evaluate health leadership and governance, including within developed countries [77]. However, as Donald Berwick (ex-administrator of the U.S. Center for Medicare and Medicaid Services) advocates, knowledge and understanding of developing country efforts can stimulate and inform health care debates in developed countries [6]. One such example is from the State of Ceará in Brazil, where innovative approaches in health leadership and management have played an important role in improving the health outcomes for Brazilians [78]. Future country cooperation can focus on knowledge exchange and the development of accepted best practices in these critical areas.

## Discussion

The core purpose of our research was to harness health systems insights to build a business case for international cooperation between developed and developing countries with a focus on the partnership-based approach. To our knowledge, no previous efforts have attempted to review benefits that developing countries may provide to developed countries. Our research findings not only confirm the existence of such benefits, but also showcase the innovative power of these experiences.

Presently, evidence-based insights on the benefits from health partnerships consist largely of soft benefits being accrued by developed country partners. Further benefits arising from the phenomenon of reverse innovation are possible in the future if opportunities are created for combined learning across health systems. Stories that anchor developing country successes can offer preliminary insights on international cooperation, including through various partnerships, collaborations, and exchanges. This evidence is seen across every part of the WHO Health Systems Framework (see Table 2 for key areas).

**Table 2:** Ten areas of health care where developed countries have the most to learn from the developing world

1	Providing services to remote areas
2	Skills substitution
3	Decentralisation of management
4	Creative problem-solving
5	Education in communicable disease control
6	Innovation in mobile phone use
7	Low technology simulation training
8	Local product manufacture
9	Health financing
10	Social entrepreneurship

There is no guarantee that knowledge and innovations from developing country experiences are necessarily appropriate for, or will transfer to, developed countries. Studying the diffusion of knowledge and innovations from developing to developed countries is a complex field outside the scope of this paper. Notwithstanding the numerous enablers and barriers to the diffusion process, the examples cited in our paper are meant to promote context-specific learning and collaboration that is reflective of the various social, economic, and political factors that influence health system development.

pt?>The phenomenon of a combined developed-developing country learning process can potentially generate effective solutions for health. Health partners who appreciate the reverse flow of knowledge and expertise have their business cases made out for them: not only do partnerships have an important role to play in balancing the mutuality of benefits, but they can also enable a sustainable two-way flow between countries to promote truly global solutions to today’s health challenges.

## Conclusions

A strong commitment to valuing different forms of knowledge is required to promote learning that challenges and rethinks traditional practice within global systems. Blending global knowledge with on-the-ground innovations from developing countries will undoubtedly transform future modes of international cooperation and any benefits accrued therefrom. However, our understanding of innovation diffusion processes between developing to developed countries is fragile, and the existing literature on this phenomenon limited. How can health innovations designed for a developing country setting be best made applicable to a developed country setting? What are the enablers and barriers to health innovation diffusion from developing to developed countries? How can largely unreported experiences by developing countries be synthesized for the global knowledge pool? An urgent next step in this complex inter-connected research agenda is to develop a standardized method for reporting the flow of health system benefits from developing to developed countries. This is perhaps the single most important component in planning for, and realizing, the full potential of international cooperation.

## Competing interests

The authors declare that they have no competing interests.

## Authors’ contributions

SBS and VD conceptualized the study and wrote the first version of the manuscript. PR, JS, JDH, RG, JC, SBN, and EK assisted in the conceptualization of the paper. SBS, VD, and DP guided the data analysis and contributed to the writing and revising of the manuscript. LD provided critical review of the paper. All authors commented on and helped with manuscript revision. All authors have read and approved the final version.

## Declaration

WHO takes no responsibility for the information provided or the views expressed in this report.

## Supplementary Material

Additional file 1 **Comprehensive search strategy employing MeSH terms and questions for critical appraisal.** (DOC 59 kb) Click here for file
