# Supplementary material for: Developed-developing country partnerships: Benefits to developed countries?
Source: Global Health. 2012 Jun 18;8:17. doi: 10.1186/1744-8603-8-17 (PMC3459713; doi:10.1186/1744-8603-8-17)
Supplement: Additional file 1 — Comprehensive search strategy employing MeSH terms and questions for critical appraisal. (DOC 59 kb) [file 1744-8603-8-17-S1.doc]

| **Appendix*:*** Comprehensive search strategy employing MeSH terms and their combinations | | |
| --- | --- | --- |
| **Set** | **PubMed** | **Detailed search strategy** |
| 1 | Health care quality, access and evaluation | Health Care Quality, Access, and Evaluation[Mesh] |
| 2 | International cooperation | International Cooperation[Mesh] |
| 3 | Hospitals | Hospitals[Mesh] |
| 4 | Learning | Learning[Mesh] |
| 5 | (Africa OR Asia) AND (Europe OR North America) | (("Africa"[Mesh] OR "Asia"[Mesh]) ) AND (("Europe"[Mesh] OR "North America"[Mesh])) |
| 6 | Sets 1 AND 2 | Health Care Quality, Access, and Evaluation[Mesh] AND International Cooperation[Mesh] |
| 7 | Sets 1 AND 3 | Health Care Quality, Access, and Evaluation[Mesh] AND Hospitals[Mesh] |
| 8 | Sets 1 AND 4 | Health Care Quality, Access, and Evaluation[Mesh] AND Learning[Mesh] |
| 9 | Sets 1 AND 5 | Health Care Quality, Access, and Evaluation[Mesh] AND (("Africa"[Mesh] OR "Asia"[Mesh]) )  AND (("Europe"[Mesh] OR "North America"[Mesh])) |
| 10 | Sets 2 AND 3 | International Cooperation[Mesh] AND Hospitals[Mesh] |
| 11 | Sets 2 AND 4 | International Cooperation[Mesh] AND Learning[Mesh] |
| 12 | Sets 2 AND 5 | International Cooperation[Mesh] AND (("Africa"[Mesh] OR "Asia"[Mesh]) ) AND (("Europe"[Mesh] OR "North America"[Mesh])) |
| 13 | Sets 3 AND 4 | Hospitals[Mesh] AND Learning[Mesh] |
| 14 | Sets 3 AND 5 | Hospitals[Mesh] AND (("Africa"[Mesh] OR "Asia"[Mesh]) ) AND (("Europe"[Mesh] OR "North America"[Mesh])) |
| 15 | Sets 4 AND 5 | Learning[Mesh] AND (("Africa"[Mesh] OR "Asia"[Mesh]) ) AND (("Europe"[Mesh] OR "North America"[Mesh])) |
| 16 | Sets 1 AND 2 AND 3 AND benefit | Health Care Quality, Access, and Evaluation[Mesh] AND International Cooperation[Mesh] AND Hospitals[Mesh] AND benefit |
| 17 | Sets 1 AND 2 AND 4 AND benefit | Health Care Quality, Access, and Evaluation[Mesh] AND International Cooperation[Mesh] AND Learning[Mesh] AND benefit |
| 18 | Sets 1 AND 2 AND 5 AND benefit | Health Care Quality, Access, and Evaluation[Mesh] AND International Cooperation[Mesh] AND (("Africa"[Mesh]  OR "Asia"[Mesh]) ) AND (("Europe"[Mesh] OR "North America"[Mesh])) AND benefit |
| 19 | Sets 1 AND 3 AND 4 AND benefit | Health Care Quality, Access, and Evaluation[Mesh] AND Hospitals[Mesh] AND Learning[Mesh] AND benefit |
| 20 | Sets 1 AND 3 AND 5 AND benefit | Health Care Quality, Access, and Evaluation[Mesh] AND Hospitals[Mesh] AND (("Africa"[Mesh] OR "Asia"[Mesh]) ) AND (("Europe"[Mesh] OR "North America"[Mesh])) AND benefit |
| 21 | Sets 1 AND 4 AND 5 AND benefit | Health Care Quality, Access, and Evaluation[Mesh] AND Learning[Mesh] AND (("Africa"[Mesh] OR "Asia"[Mesh]) ) AND (("Europe"[Mesh] OR "North America"[Mesh])) AND benefit |
| 22 | Sets 2 AND 3 AND 4 AND benefit | International Cooperation[Mesh] AND Hospitals[Mesh] AND Learning[Mesh] AND benefit |
| 23 | Sets 2 AND 3 AND 5 AND benefit | International Cooperation[Mesh] AND Hospitals[Mesh] AND (("Africa"[Mesh] OR "Asia"[Mesh]) ) AND (("Europe"[Mesh]  OR "North America"[Mesh])) AND benefit |
| 24 | Sets 2 AND 4 AND 5 AND benefit | International Cooperation[Mesh] AND Learning[Mesh] AND (("Africa"[Mesh] OR "Asia"[Mesh]) ) AND (("Europe"[Mesh]  OR "North America"[Mesh])) AND benefit |
| 25 | Sets 3 AND 4 AND 5 AND benefit | Hospitals[Mesh] AND Learning[Mesh] AND (("Africa"[Mesh] OR "Asia"[Mesh]) ) AND (("Europe"[Mesh]  OR "North America"[Mesh])) AND benefit |
| 26 | Sets 1 AND 2 AND 3 AND 4 | Health Care Quality, Access, and Evaluation[Mesh] AND International Cooperation[Mesh] AND Hospitals[Mesh]  AND Learning[Mesh] |
| 27 | Sets 1 AND 2 AND 3 AND 5 | Health Care Quality, Access, and Evaluation[Mesh] AND International Cooperation[Mesh] AND Hospitals[Mesh]  AND (("Africa"[Mesh] OR "Asia"[Mesh]) ) AND (("Europe"[Mesh] OR "North America"[Mesh])) |
| 28 | Sets 1 AND 2 AND 4 AND 5 | Health Care Quality, Access, and Evaluation[Mesh] AND International Cooperation[Mesh] AND Learning[Mesh]  AND (("Africa"[Mesh] OR "Asia"[Mesh]) ) AND (("Europe"[Mesh] OR "North America"[Mesh])) |
| 29 | Sets 1 AND 3 AND 4 AND 5 | Health Care Quality, Access, and Evaluation[Mesh] AND Hospitals[Mesh] AND Learning[Mesh]  AND (("Africa"[Mesh] OR "Asia"[Mesh]) ) AND (("Europe"[Mesh] OR "North America"[Mesh])) |
| 30 | Sets 2 AND 3 AND 4 AND 5 | International Cooperation[Mesh] AND Hospitals[Mesh] AND Learning[Mesh] AND (("Africa"[Mesh]  OR "Asia"[Mesh]) ) AND (("Europe"[Mesh] OR "North America"[Mesh])) |
| 31 | Sets 1 AND 2 AND 3 AND 4 AND 5 | Health Care Quality, Access, and Evaluation[Mesh] AND International Cooperation[Mesh] AND Hospitals[Mesh]  AND Learning[Mesh] AND (("Africa"[Mesh] OR "Asia"[Mesh]) ) AND (("Europe"[Mesh] OR "North America"[Mesh])) |

APPENDIX: Questions asked during the appraisal process

1. Is this article relevant to our research question?
2. Does this article focus on health system improvement?
3. Does the study add anything new?
4. Does the author explicitly conclude that there is a case for developed country learning?
5. Does the author implicitly refer to combined learning for developed and developing countries?
6. Has the author personally benefitted from a developed-developing country partnership?
7. Is the author relaying information on benefits from developed-developing country partnerships?
8. Were there any statistical analyses performed in the study and does the data justify the conclusion?
9. Are there any conflicts of interest?
